# Prevalence of impaired foot function in baseball players with and without disabled throwing shoulder/elbow: a case–control study

**DOI:** 10.1038/s41598-024-60513-9

**Published:** 2024-05-02

**Authors:** Hideaki Nagamoto, Shimpei Takahashi, Takumi Okunuki, Kazuki Wakamiya, Toshihiro Maemichi, Daisuke Kurokawa, Takayuki Muraki, Hiroyuki Takahashi, Nobuyuki Yamamoto, Toshimi Aizawa, Tsukasa Kumai

**Affiliations:** 1 Graduate School of Sport Sciences, Waseda University, Tokorozawa, Japan; 2 Department of Orthopaedic Surgery, Tohoku University, Sendai, Japan; 3 Department of Physical Medicine and Rehabilitation, Tohoku University Graduate School, Sendai, Japan; 4 Japan Society for the Promotion of Sciences, Tokyo, Japan; 5 Institute of Life Innovation Studies, Toyo University, Tokyo, Japan; 6 Department of Orthopaedic Surgery, Japan Community Health Care Organization Sendai Hospital, Sendai, Japan; 7 Department of Orthopaedic Surgery, Kesen-Numa City Hospital, Kesen-Numa, Japan; 8 Specified Nonprofit Organization, Network for Sports Medicine and Science, Sendai, Japan; 9 Faculty of Sport Sciences, Waseda University, 2-579-15, Mikajima, Tokorozawa, Saitama 359-1192 Japan

**Keywords:** Medical research, Risk factors

## Abstract

Prevalence of impaired foot function among baseball players with and without a disabled throwing shoulder/elbow was investigated. The study included 138 male players. Players who had previously complained of shoulder/elbow pain during throwing motion were defined as the players with a history, and those who experienced shoulder/elbow pain during the examination were defined as having the injury. Foot function was evaluated by foot “rock paper scissors” movements and floating toes. Their prevalence was assessed and the relationships between players with and without the injuries were statistically analyzed. The prevalence of players with a history and injury was 27% and 7%, respectively. The prevalence of impaired foot function on the non-throwing side among players with injury was significantly higher than those without (60% vs. 28%, *P* < 0.001) and higher tendency on the throwing side than those without (60% vs. 32%). Regarding floating toes, players with a relevant history showed a significantly higher prevalence on the throwing side than those without (49% vs 28%, *P* < 0.001) and higher tendency on the non-throwing side than those without (49% vs 32%). Players with disabled throwing shoulder/elbow have a significantly higher prevalence of impaired foot function and floating toes than players without it.

## Introduction

The foot is a composite structure of bones, ligaments, tendons and muscles^1^. For humans, they work as to support the weight, to absorb shock and impact from the surface, and create the force to push the body forward. Although its structure is small, they work as a base to support and maintain body balance. Thus, slight biomechanical changes influence its functions, and result in changes to postural-control strategies^2^. Anatomically, foot can be divided into 3 parts: forefoot, midfoot, and hindfoot. Among those, forefoot has been regarded as an extremely important part, as its functions affect the ability to stand firmly on the ground, and to stabilize the body during standing and walking or running^3^. Furthermore, especially among the forefoot, toes are important component: besides supporting and ensuring the stability of the body, they play a pivotal role in maintaining the balance of the body, and initiation of walking^3–6^. To fulfill the functions of the toes, functional skills of the intrinsic foot muscles, which also relates to maintaining and supporting the medial longitudinal arch is essential^7–10^. Previous studies have revealed that function of the intrinsic foot muscles and medial longitudinal arch relates to overuse injuries^11,12^. Therefore, to evaluate the foot function focusing on the toes among those with overuse injuries may provide useful information to reveal its relationships.

Pitching motion requires sequential and integrated motion of the whole body, from the lower extremity through the trunk to the upper extremity^13^. During pitching motion, efficient transfer of the ground reaction force is essential, which requires proper function of the lower extremity. A recent report have revealed the importance of the function of the lower extremity during throwing motion^14^. The study revealed that youth baseball players with disabled throwing shoulder and elbow tended to show positive in inability to perform deep squat test. This test has been developed as a screening test to evaluate the function of the lower extremity, including the flexibility of the ankle dorsiflexion. Another report has advocated the importance of the foot during the throwing motion^15^. Since the foot is the most distal segment of the lower extremity, it must function as a stable, yet dynamic base of support. It also serves as the base and main force generator of the kinetic chain and the initial point of the pitching motion. Despite the importance of lower extremity function has been advocated in pitching motion, only one recent retrospective study have reported about the foot function and pitching motion^16^. This retrospective study revealed that the players with disabled throwing shoulders and elbows showed high rates of impaired foot function and floating toes in both feet^16^. This suggests the importance of assessing foot function among baseball players and indicates that these pathologies may be related to the occurrence of throwing injuries, which is one of the overuse injuries. However, this study only reported the foot function and floating toes among players with disabled throwing shoulders and elbows and did not provide a comparison with players without this injury. To prove its high prevalence of impaired foot function and floating toes among players with disabled throwing shoulder and elbow, a comparison with those without the injury was necessary along with the overall prevalence of those among the youth baseball players.

Therefore, in this study, we aimed to investigate the prevalence of impaired foot function and floating toes among youth baseball players, and those among with and without disabled throwing shoulder/elbows. We hypothesized that the prevalence of impaired foot function and floating toes among baseball players would be lower than that in the previous report, and that the prevalence would be higher among players with a disabled throwing shoulder/elbow compared to those without it.

## Methods

**Figure 1:**
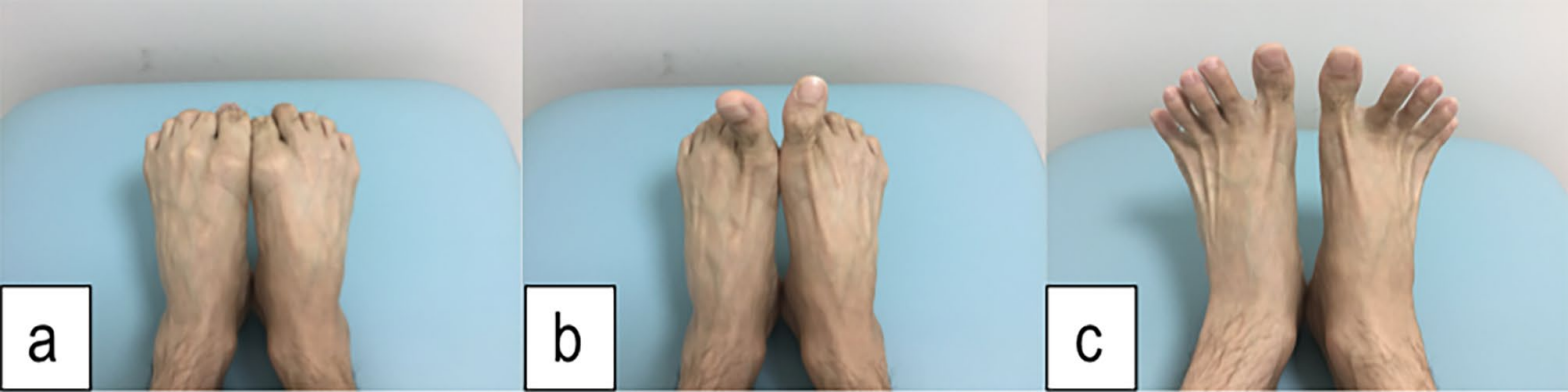
Sample figure of the foot “rock paper scissors” movements. (**a**) “rock”, (**b**) “scissors”, and (**c**) “papers”.

**Figure 2:**
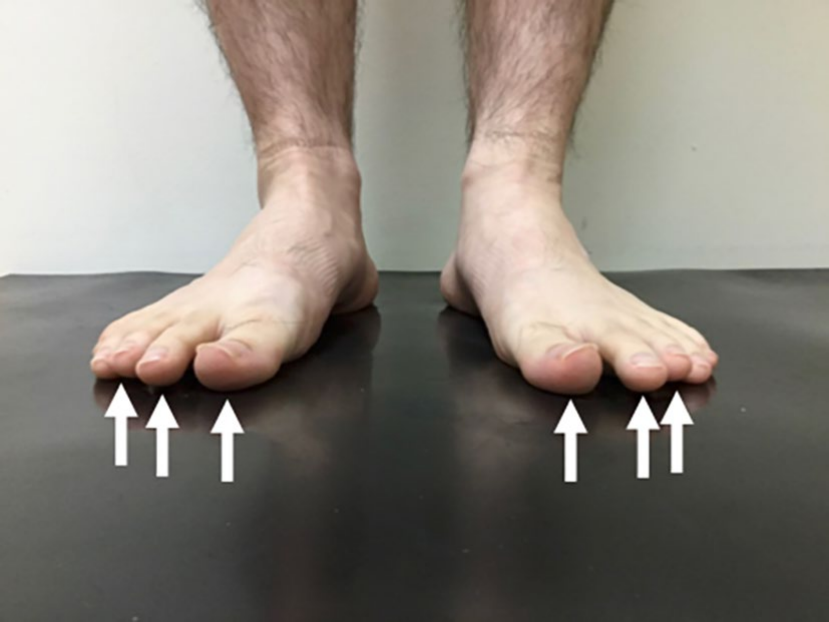
Sample image of floating toes. Toes indicated by arrows are floating toes.

This study was designed as case-controlled study and was approved by the institutional review board of our institution (IRB number 2021-185). The study was conducted in accordance with the 1964 Declaration of Helsinki and its later amendments or comparable ethical standards. The subjects were recruited from the Japanese professional baseball club’s baseball academy and was conducted as part of a preseason medical examination of the team. As the study was designed as a case-controlled study, age and sex could be the confounding factors. Hence, this study was conducted in a young male baseball teams’ preseason medical examination of a single generation to uniform the participants as possible. The purpose of the study and its potential risks were explained to the players’ parents or guardians and after which written informed consent was obtained from them. Power analysis was conducted to calculate the sample size using G-power 3.1 software (Heinrich-Heine-Universitat Dusseldorf, Germany), with the power of 0.8 at a significance level of 0.05 and the effect size of 0.3 being set. The calculation revealed that the required sample size was 88. We have decided beforehand to include all the subjects even if the number of the subjects exceeded the required sample size to avoid the selection bias. In addition, since the study was conducted as part of the preseason medical examination of a single baseball academy, providing the obtained data to the participants along with the team was essential, which lead to analyzing the data of all subjects. To comply with the results of the calculation and to analyze the whole team, the study included 139 young male baseball players. Participants who were unable to undergo evaluation of foot function or had a history of foot surgery were excluded from the study. Prior to the examination, a questionnaire was handed out asking about the participants’ dominant hand, and experience of shoulder and elbow pain of the throwing side during or after playing baseball. Age and the age when they began playing baseball was also included in the questionnaire to calculate the experiencing years of playing baseball. None of the players with shoulder or elbow pain in the past answered that they had quit a game or practice due to the pain. Players who complained of shoulder/elbow pain in the past were defined as a players with a history of disabled throwing shoulder/elbow, and those who experienced shoulder/elbow pain during the examination were defined as the players with an injury^17,18^. All participants underwent a physical examination by the senior author, followed by an evaluation of the foot function. Foot function was evaluated by foot “rock paper scissors” movements according to previous reports^5,16,19^ (Fig. 1). Foot “rock paper scissors” have been introduced as a training and evaluating method of the intrinsic foot muscles, which is easy to learn^19^. Players lay relaxed on a table and were instructed to flex all the toes to create a “rock” while maintaining the ankle in the plantar flexion position. After creating the “rock,” the players were instructed to flex their toes beside the hallux and extend the big toe to create “scissors.” To complete the evaluation, the players were instructed to extend and spread all their toes to create “paper.” Impaired foot function was defined as an inability to perform or create the above defined posture of any one of the three movements^5,16^. Floating toes were evaluated with the players standing on a solid mat on both feet, postured in a static upright position^3,16,20,21^ (Fig. 2). The players were instructed to keep their feet in shoulder-width apart while gazing at a marker 2 m ahead at eye level. Floating toes was defined if none of the toes made contact with the mat in the standing posture^3,16^. It was determined by visual inspection along with sliding the paper underneath the toes to check the clearance.

Besides the above mentioned foot function, range of motion (ROM) measurements of the lower extremity according to the previous literatures were also conducted as part of the preseason medical examination^18,22^. All the measurements were conducted using a standard goniometer while having the player layed supine on a table. Hip internal and external rotation ROM was measured according to the past literatures^18,23,24^. Players were requested to maintain their hips and knees of the measuring side flexed at 90°. An assistant stabilized the pelvis while the measurer held the lower leg of the player. The measurer passively rotated the hip internally and externally. The stable arm of the goniometer was aligned with the axis of the body, while the mobile arm was aligned to the shaft of the tibia. The angle between the arms were recorded. The straight leg raising test (SLRT) angle was measured to assess the tightness of the hamstrings following the past report^24^. The lower leg of the measuring side was raised manually by the measurer while maintaining the both of the knees in extended position. The angle measured by the goniometer between the long axis of the lower legs (mobile arm aligned along the long axis of the raised lower leg and stable arm aligned along the long axis of the contralateral leg) were defined as the SLRT angle. Ankle dorsiflexion and plantar flexion were measured following the previously described literatures^25,26^. Both of the measurements were conducted in knee extended position and measured passively. The stable arm was aligned along the medial border of the tibia and the mobile arm was aligned with the medial aspect of the foot after palpating the navicular tuberosity as the landmark. The measurer passively dorsiflexed or plantar flexed while maintaining the foot in a neutral position of inversion and eversion. The angle between the arms were then measured and both of the angles were calculated from the vertical neutral position.

The demographic data including age, years of baseball experience, height, and weight were compared between the players with and without the history of injury and with and without the injury. The prevalence of the impaired foot function and floating toes were analyzed from the results of the physical examination. Players with disabled throwing shoulder/elbow was analyzed from the answers of the questionnaire and physical examination. The relationship between prevalence of impaired foot function and floating toes and the prevalence of disabled throwing shoulders/elbows was analyzed by chi-square test. For the comparison of the range of motion measurements, Mann–Whitney U test was used for the analysis. All the statistical analyses were performed using JMP Pro 15 software (SAS Institute, Cary, NC, USA). The results were considered statistically significant at *P* value < 0.05.

### Ethics approval

The study was approved by the Ethics Review Committee on Research with Human Subjects of Waseda University (IRB number 2021-185).

### Consent to participate

Parents or the guardians, along with the players, provided written informed consent after the purpose, methods, and ethical considerations of the study were explained.

## Results

**Figure 3:**
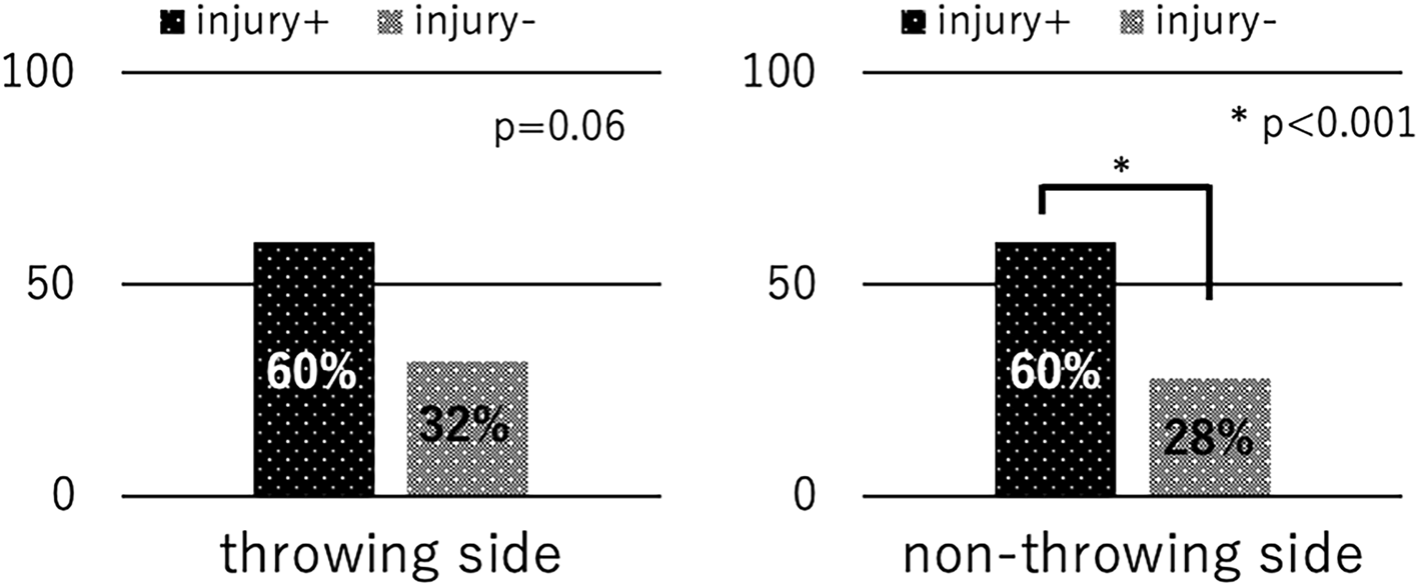
Relationship between injury to the throwing shoulder/elbow and impaired foot function.

**Figure 4:**
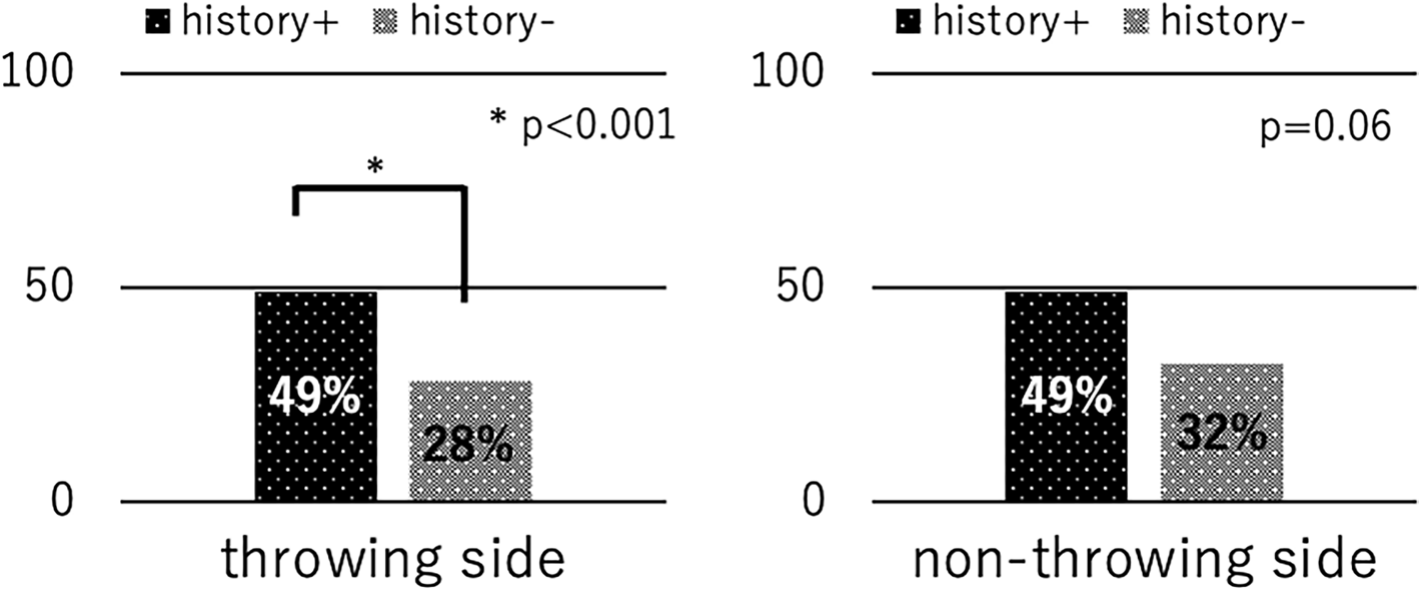
Relationship between a history of disabled throwing shoulder/elbow and floating toes.

A total of 138 male young baseball players (average age of 11.2 ± 0.7 years, range 10–12 years) met the inclusion criteria and were evaluated in the analysis. Average height, weight, and years of baseball experience of the participants were 149.1 ± 7.9 cm (range 130–170 cm), 41.8 ± 8.3 kg (range 25–65 kg), and 3.7 ± 1.4 years (range 1–6 years), respectively. These variables did not differ between any of the comparison groups except for the comparison between players with a history of disabled throwing shoulder/elbow. Players with history had slightly longer experience of playing baseball compared with those without the history (4.1 years vs. 3.5 years, *P* = 0.04). One player was excluded due to incomplete questionnaire. Among them, 121 players were right-handed, and 17 were left-handed. The prevalence of the players with a history of disabled throwing shoulder/elbow was 27% (n = 37), which included 14 players with shoulder pain, 18 players with elbow pain, and five experiencing pain in both regions. The prevalence of the players with injury was 7% (10 players), which included two players with shoulder pain, seven players with elbow pain, and one complaining of pain in both regions. The characteristics of the study participants are presented in Table 1. The overall prevalence of impaired foot function of the throwing and non-throwing sides was 34% (47 players) and 30% (42 players), respectively. The prevalence of impaired foot function on the non-throwing side among players with injury was significantly higher than that in those without injury (60% vs 28%, *P* < 0.001) (Fig. 3). Players with a history of injury tended to have a higher prevalence of impaired foot function on the throwing side than those without a history of injury (60% vs. 32%, *P* = 0.06). The overall prevalence of floating toes was 33% (46 players) and 36% (50 players). Players with a history of injury showed a significantly higher prevalence of floating toes on the throwing side than those without a history of injury (49% vs 28%, *P* < 0.001). Players with a history of injury tended to have a higher prevalence of floating toes on the non-throwing side than those without a history of injury (49% vs 32%, *P* = 0.06) (Fig. 4).

**Table 1:** Characteristics of the participants.

Variables	Age (years)	Years of baseball experience	Height (cm)	Weight (kg)	Right-handed	Left-handed
Overall	11.2 ± 0.7	3.7 ± 1.4	149.1 ± 7.9	41.8 ± 8.3	121	17
With history of injury	11.2 ± 0.6	4.1 ± 1.3*	149.4 ± 6.2	42.3 ± 8.4	32	5
Without history of injury	11.2 ± 0.7	3.5 ± 1.4*	149.0 ± 8.4	41.6 ± 8.3	89	12
With injury	11.1 ± 0.7	3.8 ± 1.2	146.7 ± 6.7	40.6 ± 6.0	7	3
Without injury	11.2 ± 0.7	3.7 ± 1.4	149.3 ± 7.9	41.9 ± 8.5	114	14

*Denotes statistically significant.

All of the ROM measurements were not statistically significant between those with or without floating toes or impaired foot function, except for the ankle dorsiflexion angle of the throwing side between those with and without floating toes (Tables 2 and 3).

**Table 2:** Relationship between range of motion and floating toes, by sides and movements.

Side	Trait	With floating toes	Without floating toes	*P*
Throwing side	Hip internal rotation	34.0 ± 1.6°	37.1 ± 1.1°	0.11
	Hip external rotation	46.1 ± 0.9°	48.9 ± 1.2°	0.14
	SLRT	61.6 ± 1.1°	62.1 ± 0.7°	0.74
	Ankle dorsiflexion	29.2 ± 0.9°	28.4 ± 0.6°	0.44
	Ankle plantar flexion	51.3 ± 1.0°	54.2 ± 0.7°	0.01*
Non-throwing side	Hip internal rotation	38.4 ± 1.5°	37.7 ± 1.2°	0.71
	Hip external rotation	47.1 ± 1.4°	46.3 ± 1.0°	0.64
	SLRT	63.1 ± 1.0°	63.1 ± 0.8°	0.98
	Ankle dorsiflexion	28.3 ± 0.7°	28.6 ± 0.6°	0.76
	Ankle plantar flexion	52.5 ± 0.9°	53.5 ± 0.7°	0.36

*SLRT* straight leg raising test.

Symbol ° stands for degrees.

*Denotes statistically significant.

**Table 3:** Relationship between range of motion and impaired foot function, by sides and movements.

Side	Trait	With impaired foot function	Without impaired foot function	*P*
Throwing side	Hip internal rotation	38.2 ± 1.5°	35.0 ± 1.1°	0.09
	Hip external rotation	46.3 ± 1.2°	48.0 ± 0.9°	0.27
	SLRT	62.3 ± 1.0°	61.7 ± 0.7°	0.62
	Ankle dorsiflexion	28.2 ± 0.9°	28.9 ± 0.6°	0.52
	Ankle plantar flexion	53.7 ± 1.0°	53.0 ± 0.7°	0.57
Non-throwing side	Hip internal rotation	40.0 ± 1.7°	37.1 ± 1.1°	0.15
	Hip external rotation	46.8 ± 1.5°	46.5 ± 1.0°	0.89
	SLRT	62.4 ± 1.1°	63.4 ± 0.7°	0.42
	Ankle dorsiflexion	28.6 ± 0.8°	28.4 ± 0.5°	0.89
	Ankle plantar flexion	54.2 ± 1.0°	52.7 ± 0.6°	0.21

*SLRT* straight leg raising test.

Symbol ° stands for degrees.

## Discussion

Overall, the results of this study showed that players with a disabled throwing shoulder/elbow had a significantly higher prevalence of impaired foot function and floating toes on both sides than players without a disabled throwing shoulder/elbow. This study is the first to directly compare foot function and floating toes between players with and without a disabled throwing shoulder or elbow. Foot “rock paper scissors” movement has been introduced as a method to assess foot function, particularly of the foot’s intrinsic muscles and its functional skill^5,6,19^. The intrinsic foot muscles provide stability to create propulsive force^1^, and thus have great importance in postural-control^7^ and are crucial in maintaining and supporting the medial longitudinal arch^8–10,19^. Proper function of the medial longitudinal arch as well as the intrinsic foot muscles is important, as several investigators have reported a relationship with overuse injuries^11,12^. Thus, assessing the foot function focusing on intrinsic foot muscles among baseball players is necessary given the recent report of impaired foot function among players with disabled throwing shoulder/elbow^16^. Although the intrinsic foot muscles are relatively small muscles with small moment arms and cross-sectional area^27^, several researchers have proposed that their function relates with sports performance^28^. Further studies revealing the relationship between pitching performance and foot function need to be conducted in the future.

Past studies have revealed a relationship between decreased toe flexor strength and toe deformities^29^, especially in those with floating toes^20^. The contact between the toes and the ground enables pressure to be exerted against the ground, allowing humans to maintain balance^30^ and perform many essential activities in daily living, such as walking and standing^31^. Furthermore, toe flexor muscle strength is not only related to daily activities but also contributes to physical performance among athletes. Several studies have shown that toe flexor strength is positively associated with the performance of sporting activities^32,33^. A decrease in toe flexor strength can cause trouble during movement^34^, and is reportedly associated with impaired physical performance among athletes^35^. Future studies need to be conducted to reveal if improvement in foot function can decrease the incidence of disabled throwing shoulder/elbow.

The results of our study showed that the prevalence of floating toes among young baseball players was 33 to 36%, which was similar to the 40% prevalence noted in previous reports^21,36^. On the other hand, the prevalence of floating toes among the players with disabled throwing shoulder/elbow was 49%, which was lower than that in a previous study^16^. This may be explained by the differences in participants between studies; for example, the study by Nagamoto et al. only included those who visited the outpatient clinic complaining of shoulder and/or elbow pain at the time of the consultation. This peculiar background may have increased the prevalence of floating toe. Additionally, no studies have reported the prevalence of foot function impairment in the general population assessed using the foot “rock paper scissors” movement; therefore, further studies may are required to investigate this prevalence.

Among several ROM variables in lower extremity, only the ankle plantar flexion on the throwing side showed significant difference between those with and without floating toes, that those with floating toes had significantly smaller ROM of ankle plantar flexion on the throwing side. While there are no studies in the past that have revealed the ROM difference in those with and without floating toes, Soma et al. have revealed that toe grip strength significantly decreases in ankle plantar flexed position compared with when they were evaluated in neutral or dorsiflexed position^37^. They have suspected that strength decreased in ankle plantar flexed position since the bi-articular muscles related to toe flexing (i.e. flexor hallucis longus and flexor digitorum longus) shortens in plantar flexed position. In addition, suppressor to these muscles, in this case, the extensors, stretches when the ankle is plantar flexed, creating resisting tension leading to decrease in toe grip strength. It has been revealed that those with floating toes show decreased toe flexor strength and propulsive force in forward motion^20^. Therefore, due to the decreased toe flexor strength and force to provide forward motion, those with floating toes may not be able to plantar flex the ankle maximally, probably leading to significant decrease in ROM compared with those without it. From these facts and our results may show that the restricted ankle plantar flexion ROM may have a relationship between floating toes and that the future studies need to reveal if those are the cause or the result of the floating toes.

Our study had several limitations. Firstly, our results should be interpreted with caution as this study was a cross-sectional study; therefore, a cause-and-effect relationship cannot be established. Future studies should be conducted to elucidate this relationship. Although the foot “rock paper scissors” movement and floating toes indicate decreased intrinsic foot muscle or toe flexor muscle strength, the strength of the muscles was not measured directly. However, toe flexor strength is provided by a combination of the intrinsic and extrinsic foot muscles^38,39^ and cannot be differentiated between them. Furthermore, a direct method to measure intrinsic muscle strength is still controversial and has not yet been developed^40^; therefore, direct measurement of strength could not be performed. Foot shape (e.g. arch height index) was also not measured. Previous studies have shown that decrease in strength of abductor hallucis and flexor hallucis brevis did not cause a change in collapsing of the medial longitudinal arch height^41^. In addition, considering that the study was undertake as part of a large-scale preseason medical examination, which required to complete the examination without hindrance, we decided not to measure the foot shape. The average age of the participants were slightly younger than the previous report^16^. Therefore, applying the results to the same generation may need careful discretion. Future studies conducted to the same generation is essential. Number of pitches, main position, and starting age of pitching may have influenced the results, especially the experiencing the pain in shoulder and elbow. However, as the participants were primary schoolers, they played multiple positions in different teams. Therefore, asking the number of “pitches”, starting age of pitching, and position were useless. Type of shoes (sneakers or cleats) may have affected the foot function. Future studies revealing the effect of shoe type and foot function should be performed. Lastly, as the study was undertaken as part of a large-scale preseason medical examiation, completing the examination without hindrance was required, which we decided not to measure the arch height index.

## Conclusions

In conclusion, this study notified a lower prevalence of impaired foot function and floating toes among youth baseball players compared with previous report of those of baseball players with disabled throwing shoulder/elbow. Impaired foot function and floating toes were highly observed among those with disabled throwing shoulder/elbow compared to their counterparts without such disabilities. These findings suggest a potential association between impaired foot function, floating toes, and disabled throwing shoulders and elbows.

## Data Availability

The datasets used and/or analyzed during this study are available from the corresponding author on reasonable request.
